# Risk of Non‐Arteritic Anterior Ischemic Optic Neuropathy in Idiopathic Intracranial Hypertension Patients Treated with GLP‐1 Receptor Agonists

**DOI:** 10.1002/acn3.70406

**Published:** 2026-04-17

**Authors:** Faisal A. Al‐Harbi, Mohanad A. Alkuwaiti, Yazeed B. Alaql, Ahmed K. Alsaif, Ahmed A. Alessa, Meshari Ayed Alharbi, Mohammed Alfalah, Saud A. Alnaaim, Sajjad M. AlHaddad, Ahmed Y. Azzam

**Affiliations:** ^1^ College of Medicine Qassim University Buraydah Saudi Arabia; ^2^ College of Medicine Imam Abdulrahman Bin Faisal University Dammam Saudi Arabia; ^3^ College of Medicine Al‐Rayan Colleges Al‐Madinah Saudi Arabia; ^4^ Faculty of Medicine King Abdulaziz University Jeddah Saudi Arabia; ^5^ Qassim Health Cluster Qassim Saudi Arabia; ^6^ College of Medicine King Faisal University Al‐Ahsa Saudi Arabia; ^7^ Department of Endocrinology and Diabetes King Fahad Medical City (KFMC) Riyadh Saudi Arabia; ^8^ Director of Clinical Research and Clinical Artificial Intelligence, ASIDE Healthcare Lewes Delaware USA; ^9^ Division of Global Health and Public Health, School of Nursing, Midwifery and Public Health University of Suffolk Ipswich UK

**Keywords:** GLP‐1 receptor agonists, idiopathic intracranial hypertension, non‐Arteritic anterior ischemic optic neuropathy, optic atrophy, pseudotumor cerebri

## Abstract

**Introduction:**

Glucagon‐like peptide‐1 receptor agonists (GLP‐1 RAs) have demonstrated significant weight‐reducing effects and may offer benefits in idiopathic intracranial hypertension (IIH); however, recent concerns about the risk of non‐arteritic anterior ischemic optic neuropathy (NAION) have emerged. Hence, this study was designed to examine the relationship between GLP‐1 RA use and optic outcomes in IIH patients.

**Methods:**

This study used the TriNetX Global Collaborative Network to perform a retrospective propensity score‐matched cohort study. It involved GLP‐1 RA exposure status to classify adult patients with IIH. Further, it applied propensity score matching (PSM) on demographics, comorbidities, and medications. In this study, the primary outcome was incident NAION; the secondary outcome was optic atrophy. Cox proportional hazards regression, time‐stratified analyses, and multiple testing corrections were performed.

**Results:**

From 144,678 IIH patients, 15,567 matched pairs were analyzed. GLP‐1 RA use demonstrated significantly decreased NAION risk (hazard ratio [HR] 0.47, 95% confidence interval [CI] 0.24–0.93, *p* = 0.027) and optic atrophy risk (HR 0.78, 95% CI 0.64–0.94, *p* = 0.009). Time‐stratified analyses have demonstrated protective associations across all evaluated time windows. The optic atrophy finding remained significant after Bonferroni correction, while NAION remained significant after false discovery rate correction. Subgroup analysis revealed stronger protective associations in non‐diabetic patients (risk ratio 0.53, 95% CI 0.41–0.68) compared to diabetic patients (risk ratio 0.76, 95% CI 0.58–0.99).

**Conclusions:**

GLP‐1 RA utilization among IIH patients was associated with a reduced risk of NAION and optic atrophy. These findings align with the proposed pathway linking GLP‐1 RA‐induced weight reduction to lower intracranial pressure and papilledema reduction in IIH patients; residual confounding cannot be ruled out, and prospective studies are needed to confirm these associations.

## 1 Introduction

Idiopathic intracranial hypertension (IIH) manifests as an unexplained elevation of cerebrospinal fluid (CSF) pressure, mainly affecting young women with obesity [1]. It presents a significant risk for visual impairment, with non‐arteritic anterior ischemic optic neuropathy (NAION) and progressive optic atrophy representing serious complications that may lead to permanent vision loss [2, 3, 4]. Current management strategies focus on weight reduction, carbonic anhydrase inhibitors, and surgical interventions in refractory cases; however, visual outcomes remain suboptimal in a significant proportion of patients [5, 6, 7, 8].

Moreover, GLP‐1 RAs, developed initially as antihyperglycemic agents for type 2 diabetes mellitus (T2DM), have demonstrated significant weight reduction effects and cardiovascular benefits across different patient populations [9, 10, 11, 12, 13, 14, 15, 16, 17, 18]. These agents function via multiple pathways, including appetite suppression, delayed gastric emptying, and improved metabolic parameters. Given the strong association between obesity and IIH, GLP‐1 RAs have increasingly been recognized as a potentially valuable therapeutic approach for weight management in this population [19, 20, 21, 22, 23]. However, recent pharmacovigilance signals and observational studies have raised concerns regarding possible associations between GLP‐1 RA use and NAION [24, 25, 26], creating uncertainty among physicians managing IIH patients. Furthermore, the association between GLP‐1 RAs and optic nerve outcomes in IIH remains incompletely characterized. While weight reduction achieved through GLP‐1 RA therapy could reduce intracranial pressure and improve optic nerve perfusion, the possible direct vascular effects of these medications on optic nerve blood supply remain unclear. In addition, whether the reported NAION associations apply to IIH patients, who already carry elevated baseline risk for optic complications, has not been investigated [27, 28].

To address these evidence gaps, this study designed this retrospective cohort investigation to assess the relationship between GLP‐1 RA use and incident optic outcomes in IIH patients utilizing a large, federated healthcare database, TriNetX Global Database, with propensity score matching methodology.

## 2 Methods

### 2.1 Study Design and Data Source

This retrospective cohort study adhered to the Strengthening the Reporting of Observational Studies in Epidemiology (STROBE) guidelines [29, 30]. It utilized the TriNetX Global Collaborative Network, a federated healthcare research platform that includes electronic health records from over 100 million patients across more than 80 healthcare organizations worldwide. The platform provides access to de‐identified patient‐level data, including demographics, diagnoses, procedures, medications, and laboratory values [31].

The Western Institutional Review Board issued a waiver for this study's use of the TriNetX platform. This waiver applies because TriNetX operates as a federated research network wherein all accessible information, whether presented as aggregated summaries or individual‐level record extracts, consists exclusively of de‐identified patient data meeting the standards specified in Section §164.514(a) of the HIPAA Privacy Rule. In addition to that, this retrospective study qualifies for exemption from informed consent requirements. The study constitutes a secondary evaluation of pre‐existing records without any direct patient intervention or human subject interaction, utilizing data de‐identified according to Section §164.514(a) of the HIPAA Privacy Rule. A qualified expert, as outlined in Section §164.514(b)(1) of the HIPAA Privacy Rule, has provided formal attestation verifying the de‐identification methodology.

### 2.2 Study Population and Exposure Classification

This study identified adult patients aged 18 years or older with an IIH diagnosis based on the International Classification of Diseases, Tenth Revision, Clinical Modification (ICD‐10‐CM) code G93.2. Patients were classified into two cohorts based on GLP‐1 RA exposure: the exposed cohort included patients with documented prescriptions for any GLP‐1 RA including semaglutide, liraglutide, dulaglutide, exenatide, tirzepatide, or lixisenatide; the unexposed cohort included patients without GLP‐1 RA exposure. The index date was defined as the date of the first GLP‐1 RA prescription for the exposed cohort.

### 2.3 Outcome Definitions

The primary outcome was incident NAION, defined using ICD‐10‐CM code H47.01, following the coding methodology utilized in previous TriNetX‐based studies evaluating optic neuropathy outcomes. The secondary outcome was optic atrophy, defined using ICD‐10‐CM codes H47.20, H47.21, H47.22, and H47.23. Further, this study defined optic atrophy as a broad category since the TriNetX analysis platform does not provide coding for post‐NAION optic atrophy as a distinct outcome, and its outcome analysis function does not allow time‐controlled sequential event definitions. Notably, this study acknowledged this limitation in the study design. Patients with a prior diagnosis of the respective outcome before the index date were excluded from each analysis.

### 2.4 Propensity Score Matching

To minimize confounding by indication and baseline differences, this study performed one‐to‐one PSM using TriNetX built‐in statistical tools, based on the platform's available matching methodology. Propensity scores were estimated using logistic regression including age, sex, race, ethnicity, body mass index (BMI), diabetes mellitus, disorders of lipid metabolism, diseases of the circulatory system, diseases of the eye and adnexa, central nervous system medications, and ophthalmic agents. Matching was performed using the nearest‐neighbor algorithm with a caliper width of 0.1 standard deviations. Balance was assessed using standardized mean differences (SMDs); values less than 0.10 indicated adequate balance.

### 2.5 Statistical Analysis

This study utilized the built‐in analysis functions of the TriNetX platform for primary analyses, including risk ratios, odds ratios, hazard ratios from Cox proportional hazards regression, and Kaplan–Meier survival analyses. Additionally, it utilized RStudio statistical software with R version 4.4.2 for analyses beyond the TriNetX platform capabilities, including restricted mean survival time (RMST) analysis, multiple testing corrections, Type S and Type M error analyses using the Gelman‐Carlin framework, leave‐one‐out sensitivity analyses, probabilistic bias analysis with Monte Carlo simulation, and replication probability simulations. Multiple testing corrections were performed using Bonferroni, Benjamini‐Hochberg false discovery rate (FDR), and Holm step‐down methods across eight hypothesis tests. E‐values were calculated to assess significance to unmeasured confounding. Statistical significance was defined as a two‐tailed *p* < 0.05. The Cox proportional hazards model was implemented using the TriNetX platform's built‐in analysis function, which employs a conventional (unstratified) Cox regression without stratification by matched pairs, as matched‐pair identifiers are not available in the federated analysis environment. To address this limitation, the consistency of results was verified across multiple independent analytical approaches. The proportional hazards assumption was assessed using log–log survival plots derived from the Kaplan–Meier estimates. Additionally, sensitivity analyses were performed including restriction to a common 2‐year follow‐up window to address differential censoring between cohorts, and Firth‐corrected (penalized) estimates to address potential sparse‐data bias for the NAION outcome.

## 3 Results

### 3.1 Study Population and Baseline Characteristics

The study flow is presented in Figure 1. Of the 144,678 patients with IIH diagnosis, 16,673 (11.5%) had GLP‐1 RA exposure, and 128,005 (88.5%) had no GLP‐1 RA exposure. After PSM, 15,567 matched pairs were included in the analysis. Baseline demographics and characteristics before and after PSM are presented in Table 1. Before matching, significant imbalances were observed with the GLP‐1 RA cohort being older (mean 43.8 versus 38.4 year‐old), having a higher female proportion (91.2% versus 79.8%), and demonstrating significantly higher prevalence of diabetes mellitus (39.3% versus 7.1%, SMD = 0.826), lipid disorders (45.7% versus 12.9%, SMD = 0.772), and a higher BMI (41.7 versus 34.2 kg/m^2^, SMD = 0.802). After PSM, all covariates achieved adequate balance with SMD less than 0.10, except BMI, which retained residual imbalance (41.6 versus 35.6 kg/m^2^, SMD = 0.642), as demonstrated in Figure S1. The mean follow‐up for the GLP‐1 RA cohort and the non‐GLP‐1 RA cohort was 747 ± 729 and 1239 ± 1203 days, respectively.

**FIGURE 1:**
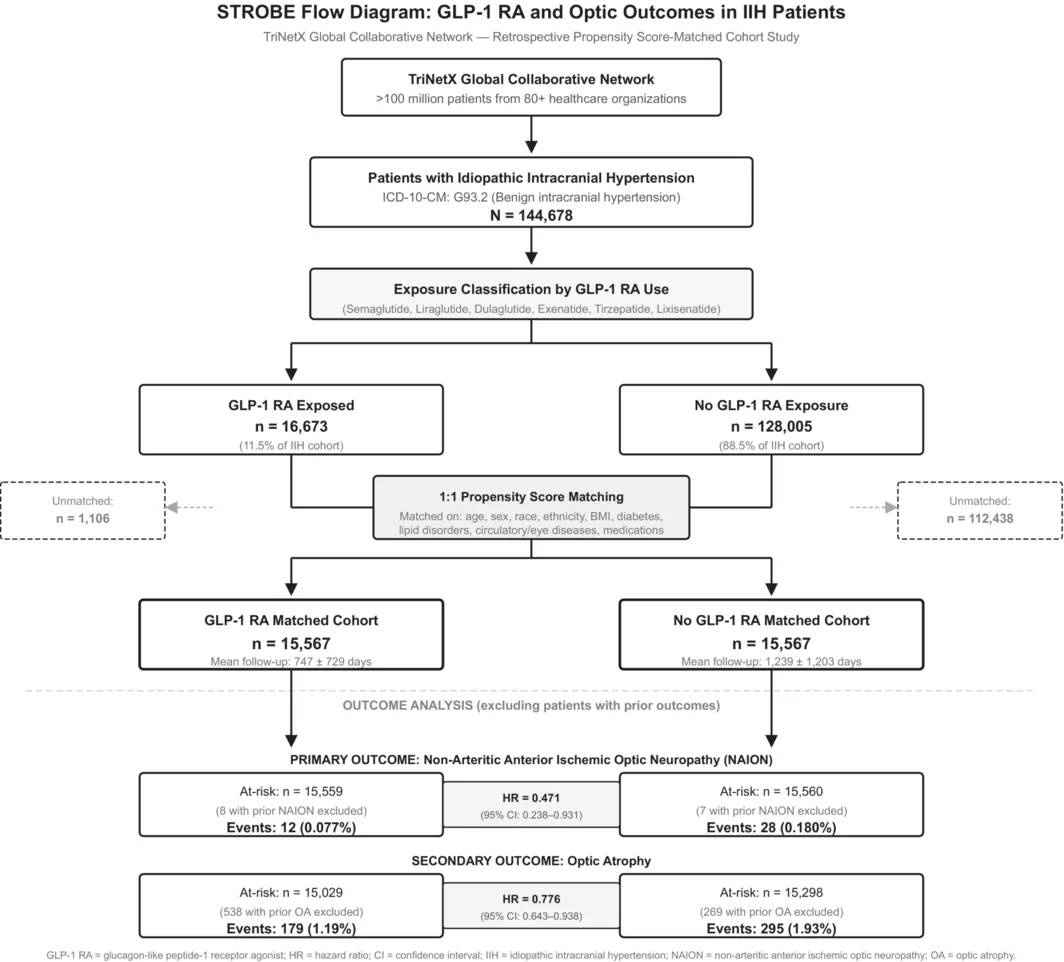
STROBE flow diagram.

**TABLE 1:** Baseline demographics and clinical characteristics before and after propensity score matching.

Characteristic	Before PSM	Before PSM	Before PSM	Before PSM	After PSM	After PSM	After PSM	After PSM
	GLP‐1 RA (*n* = 16,673)	No GLP‐1 RA (*n* = 128,005)	*p*	SMD	GLP‐1 RA (*n* = 15,567)	No GLP‐1 RA (*n* = 15,567)	*p*	SMD
Demographics								
Age, years, mean ± SD	43.8 ± 12.7	38.4 ± 16.4	< 0.001	0.369	43.5 ± 12.7	44.4 ± 15.5	< 0.001	0.068
Female sex, %	91.2	79.8	< 0.001	0.326	90.5	91.0	0.117	0.018
Comorbidities, %								
Diabetes mellitus	39.3	7.1	< 0.001	0.826	35.0	33.1	0.001	0.039
Lipid disorders	45.7	12.9	< 0.001	0.772	42.7	41.9	0.130	0.017
Circulatory system diseases	70.4	31.2	< 0.001	0.854	68.4	69.9	0.005	0.032
Eye and adnexa diseases	63.6	28.5	< 0.001	0.752	61.6	61.5	0.861	0.002
Clinical measures								
BMI, kg/m^2^, mean ± SD	41.7 ± 9.1	34.2 ± 9.4	< 0.001	0.802	41.6 ± 9.1	35.6 ± 9.6	< 0.001	0.642*
Follow‐up time								
Mean, days ± SD	760 ± 740	1301 ± 1374	< 0.001	—	747 ± 729	1239 ± 1203	< 0.001	—
Median (IQR), days	568 (782)	838 (1892)	—	—	559 (769)	888 (1625)	—	—

Abbreviations: BMI, body mass index; BP, blood pressure; CNS, central nervous system; GLP‐1 RA, glucagon‐like peptide‐1 receptor agonist; IQR, interquartile range; *p*, *p*‐value; PSM, propensity score matching; SD, standard deviation; SMD, standardized mean difference.

* Indicates inadequate balance after PSM (SMD > 0.10). Propensity score matching performed using 1:1 nearest‐neighbor matching with logistic regression on age, sex, race, ethnicity, diabetes mellitus, lipid disorders, circulatory system diseases, eye and adnexa diseases, CNS medications, ophthalmic agents, BMI, systolic BP, diastolic BP, and heart rate.

Table S1 presents ICD‐10 codes and medication classifications utilized in this study.

### 3.2 Primary and Secondary Outcomes

Primary and secondary outcome results are presented in Table 2 and visualized in Figure 2. For NAION, 12 events occurred in the GLP‐1 RA cohort (0.077%) compared to 28 events in the non‐GLP‐1 RA cohort (0.180%), resulting in an HR of 0.47 (95% CI 0.24–0.93, *p* = 0.027). This estimate should be interpreted with caution. The risk ratio was 0.43 (95% CI 0.22–0.84, *p* = 0.011) with a number needed to treat (NNT) of 973. NNT figures here reflect the observed absolute risk difference in this matched cohort. They are not treatment recommendations; this is an observational study, and causality was not established. For optic atrophy, 179 events occurred in the GLP‐1 RA cohort (1.19%) compared to 295 events in the non‐GLP‐1 RA cohort (1.93%), resulting in an HR of 0.78 (95% CI 0.64–0.94, *p* = 0.009). The risk ratio was 0.62 (95% CI 0.51–0.74, *p* < 0.001) with NNT of 136. E‐values for NAION and optic atrophy were 4.09 and 2.62, respectively.

**TABLE 2:** Primary and secondary outcomes with effect measures.

Measure	NAION (primary outcome)	Optic atrophy (secondary outcome)
Event data		
GLP‐1 RA events/at risk, *n*/*N*	12/15,559	179/15,029
No GLP‐1 RA events/at risk, *n*/*N*	28/15,560	295/15,298
GLP‐1 RA risk, %	0.077	1.191
No GLP‐1 RA risk, %	0.180	1.928
Relative effect measures		
Risk ratio (95% CI)	0.429 (0.218–0.843)	0.618 (0.514–0.743)
Odds ratio (95% CI)	0.428 (0.218–0.842)	0.613 (0.508–0.739)
Hazard ratio (95% CI)^a^	0.471 (0.238–0.931)	0.776 (0.643–0.938)
*p* (risk ratio)	0.011	< 0.001
*p* (log‐rank)	0.027	0.009
Absolute effect measures		
Risk difference per 1000	−1.03	−7.37
Absolute risk reduction per 1000	1.03	7.37
Number needed to treat (95% CI)	973 (548–4308)	136 (98–218)
Robustness metrics		
Fragility index	3^b^	66^b^
E‐value, point estimate	4.09	2.62
E‐value, confidence interval bound	1.66	2.03

Abbreviations: CI, confidence interval; GLP‐1 RA, glucagon‐like peptide‐1 receptor agonist; IIH, idiopathic intracranial hypertension; NAION, non‐arteritic anterior ischemic optic neuropathy.

^a^ Hazard Ratio from Cox proportional hazards regression designated as primary effect measure to account for differential follow‐up time between cohorts (GLP‐1 RA: mean 747 days vs No GLP‐1 RA: mean 1239 days). Risk Ratio reported as secondary measure. E‐value represents the minimum strength of association an unmeasured confounder would need with both treatment and outcome to fully explain away the observed effect; higher values indicate greater robustness to unmeasured confounding. Fragility Index represents the minimum number of event status changes required to reverse statistical significance; values ≤ 3 suggest fragile findings. Number Needed to Treat reflects the observed absolute risk difference per 1000 patients in this matched cohort and should not be interpreted as a prospective treatment estimate or causal effect.

^b^ Fragility index calculated by adding events to the GLP‐1 RA group until statistical significance is lost (Fisher's exact test). See Table S2 for detailed leave‐one‐out analysis.

**FIGURE 2:**
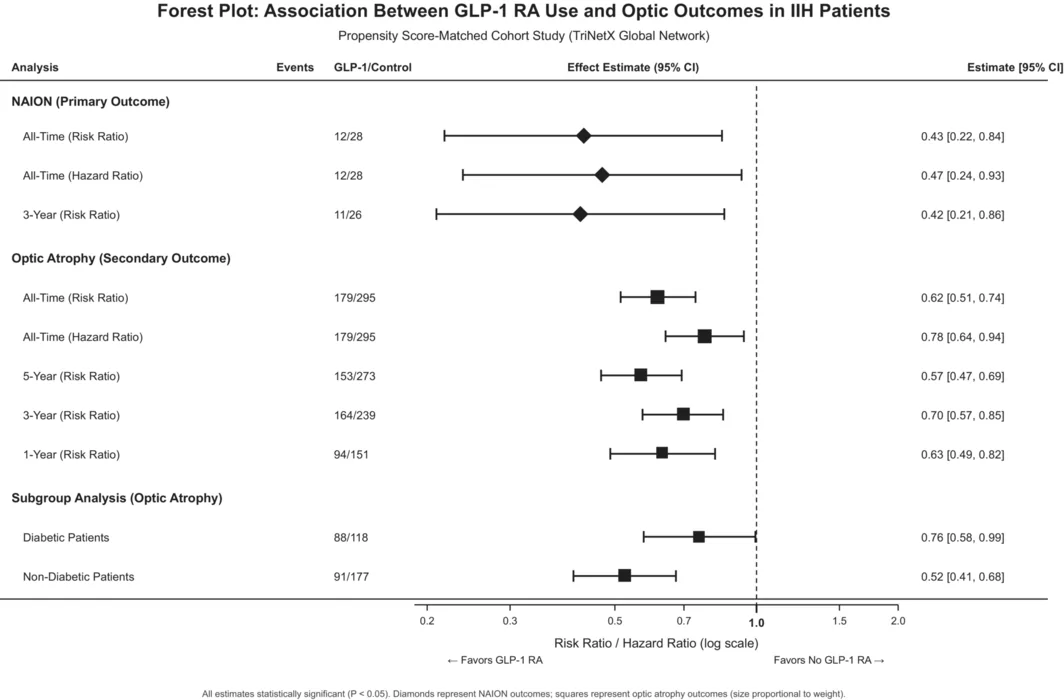
Forest plot for association between GLP‐1 RA and optic outcomes.

### 3.3 Time‐Stratified Analyses

Time‐stratified analyses are presented in Table 3 and Kaplan–Meier curves are shown in Figure 3. NAION demonstrated a protective association at the 3‐year analysis (risk ratio 0.42, 95% CI 0.21–0.86, *p* = 0.014). Optic atrophy demonstrated protective associations across all time windows: one‐year (risk ratio 0.63, 95% CI 0.49–0.82), three‐year (risk ratio 0.70, 95% CI 0.57–0.85), and five‐year (risk ratio 0.57, 95% CI 0.47–0.69), all with *p* < 0.001. RMST analysis revealed significantly longer event‐free survival in the GLP‐1 RA cohort for both NAION (ΔRMST = 0.46 days, 95% CI 0.06–0.86, *p* = 0.023) and optic atrophy (ΔRMST = 2.07 days, 95% CI 0.58–3.56, *p* = 0.006), as shown in Figure 4.

**TABLE 3:** Time‐stratified analyses and survival metrics.

Outcome and time window	GLP‐1 RA events/*N*	No GLP‐1 RA events/*N*	Risk ratio (95% CI)	Hazard ratio (95% CI)	*p*	NNT (95% CI)
NAION						
All‐time	12/15,559	28/15,560	0.429 (0.218–0.843)	0.471 (0.238–0.931)*	0.011	973 (548–4308)
3‐year	11/15,559	26/15,560	0.423 (0.209–0.856)	—	0.014	1037 (578–5044)
Optic atrophy						
All‐time	179/15,029	295/15,298	0.618 (0.514–0.743)	0.776 (0.643–0.938)*	< 0.001	136 (98–218)
5‐year	153/13,753	273/13,971	0.569 (0.468–0.693)	—	< 0.001	119 (88–181)
3‐year	164/15,029	239/15,298	0.698 (0.573–0.851)	—	< 0.001	212 (137–468)
1‐year	94/13,751	151/13,986	0.633 (0.490–0.818)	—	< 0.001	252 (162–567)
Follow‐up time	GLP‐1 RA cohort	No GLP‐1 RA cohort	Difference	—	—	—
Mean, days ± SD	747 ± 729	1239 ± 1203	−492	—	—	—
Median (IQR), days	559 (769)	888 (1625)	−329	—	—	—

Abbreviations: CI, confidence interval; GLP‐1 RA, glucagon‐like peptide‐1 receptor agonist; HR, hazard ratio; IIH, idiopathic intracranial hypertension; IQR, interquartile range; NAION, non‐arteritic anterior ischemic optic neuropathy; NNT, number needed to treat; SD, standard deviation.

* Hazard Ratio from Cox proportional hazards regression reported for All‐Time analyses only. *p*‐values correspond to Risk Ratio comparisons. Time windows defined as: 1‐year (≤ 365 days), 3‐year (≤ 1095 days), 5‐year (≤ 1825 days), All‐Time (unlimited follow‐up from index event). The protective association was consistent across all time windows for both outcomes, with effect sizes ranging from 30% to 57% risk reduction for NAION and 30% to 43% risk reduction for optic atrophy. Differential follow‐up time between cohorts (GLP‐1 RA mean 747 days vs No GLP‐1 RA mean 1239 days) supports the use of Hazard Ratio as the primary effect measure for All‐Time analyses. NNT calculated from absolute risk reduction; values indicate number of IIH patients needed to treat with GLP‐1 RA to prevent one outcome event within the specified time window. — indicates data not available.

**FIGURE 3:**
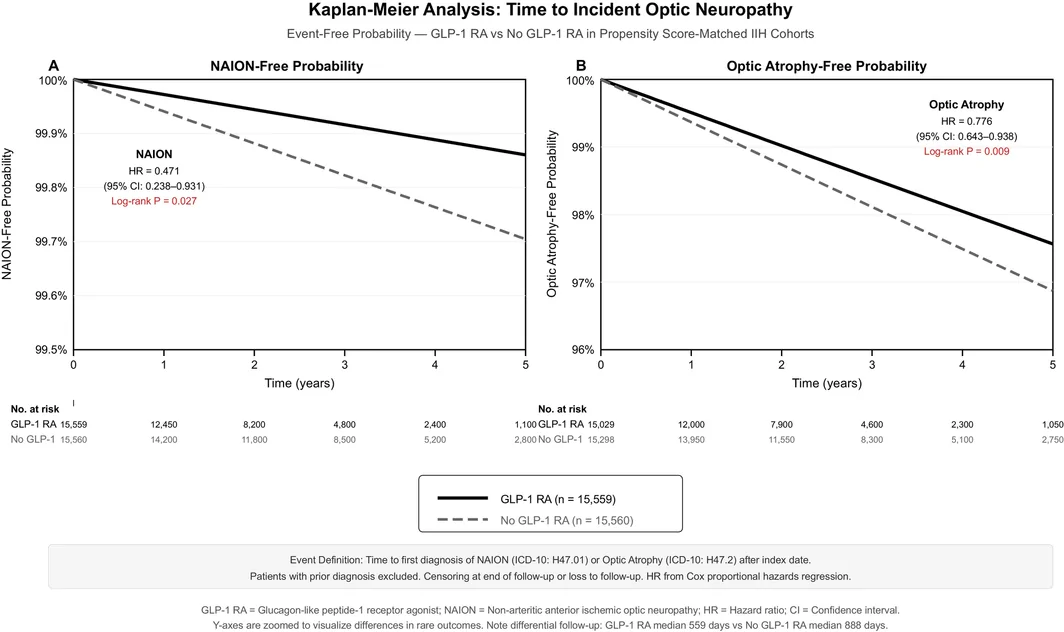
Kaplan–Meier curves.

**FIGURE 4:**
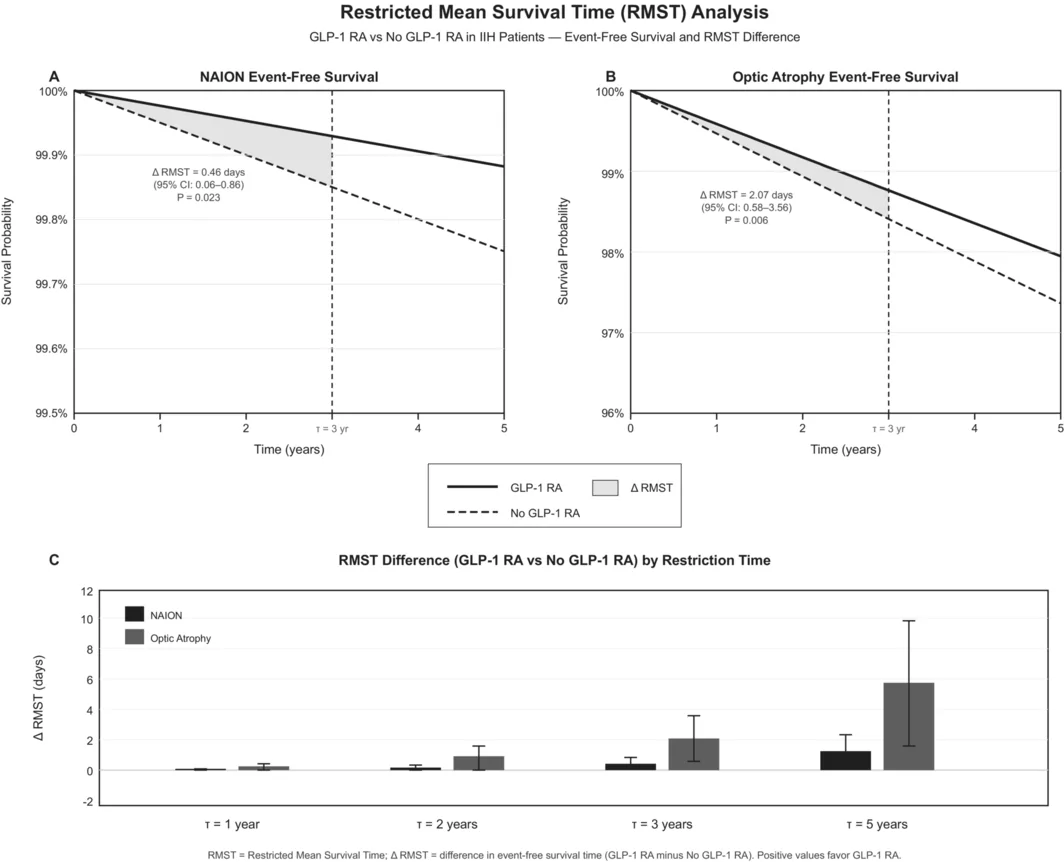
Restricted mean survival time plot.

### 3.4 Subgroup Analyses

Subgroup analyses by diabetes status for optic atrophy are presented in Table 4. The protective effect was observed in both diabetic patients (risk ratio 0.76, 95% CI 0.58–0.99, *p* = 0.045) and non‐diabetic patients (risk ratio 0.53, 95% CI 0.41–0.68, *p* < 0.001). The protective effect was stronger in non‐diabetic patients (47.5% relative risk reduction) than in diabetic patients (24.4% relative risk reduction), with a borderline significant interaction (*p* = 0.054).

**TABLE 4:** Subgroup analyses by diabetes status for optic atrophy outcome.

Measure	Overall	Diabetic subgroup	Non‐diabetic subgroup
Sample size			
GLP‐1 RA, *n*	15,029	6321	8708
No GLP‐1 RA, *n*	15,298	6408	8890
Event data			
GLP‐1 RA events/at‐risk, *n*/*N*	179/15,029	88/6321	91/8708
No GLP‐1 RA events/at‐risk, *n*/*N*	295/15,298	118/6408	177/8890
GLP‐1 RA risk, %	1.191	1.392	1.045
No GLP‐1 RA risk, %	1.928	1.841	1.991
Relative effect measures			
Risk Ratio (95% CI)	0.618 (0.514–0.743)	0.756 (0.575–0.994)	0.525 (0.408–0.675)
Odds Ratio (95% CI)	0.613 (0.508–0.739)	0.753 (0.569–0.997)	0.520 (0.402–0.672)
Relative risk reduction, %	38.2	24.4	47.5
*p*‐value	< 0.001	0.045	< 0.001
Absolute effect measures			
Risk difference per 1000	−7.37	−4.49	−9.46
Number needed to treat (95% CI)	136 (98–218)	223 (113–8834)*	106 (77–171)
Robustness metrics			
Fragility index	35	1*	48
E‐value, point estimate	2.62	1.98	3.22
E‐value, confidence interval bound	2.03	1.08	2.33
Interaction analysis			
Ratio of risk ratios (95% CI)	—	1.440 (0.993–2.089)	Reference
*p*‐value for interaction	—	0.054	—

Abbreviations: CI, confidence interval; E‐value, evidence value for unmeasured confounding; GLP‐1 RA, glucagon‐like peptide‐1 receptor agonist; ICD‐10, international classification of diseases, tenth revision; NNT, number needed to treat.

* Indicates fragile finding. The diabetic subgroup has a Fragility Index of 1, indicating that a change in outcome status for a single patient would reverse statistical significance. The wide NNT confidence interval (113–8834) reflects uncertainty in the absolute effect estimate for this subgroup. The protective effect was present in both subgroups but was significantly stronger in non‐diabetic patients (47.5% vs 24.4% relative risk reduction), with borderline significant interaction (*p* = 0.054). This pattern suggests the protective effect may be mediated by weight loss mechanisms rather than being confounded by diabetes treatment indication, as the effect is more pronounced when diabetes‐related confounding is absent. E‐value represents the minimum strength of association an unmeasured confounder would need with both treatment and outcome to fully explain away the observed effect. — indicates not applicable.

### 3.5 Multiple Testing Corrections and Sensitivity Analyses

Multiple testing corrections and sensitivity analyses are presented in Table 5 and Figure S2. After Bonferroni correction (α/8 = 0.00625), all optic atrophy analyses except the diabetic subgroup remained significant. NAION findings remained significant after FDR and Holm corrections but not after Bonferroni correction. Type S and Type M error analysis demonstrated negligible probability of sign error and minimal exaggeration ratio for both outcomes at observed effect sizes, as shown in Figure 5. The fragility index was 3 for NAION and 66 for optic atrophy.

**TABLE 5:** Multiple testing corrections and sensitivity analyses.

Analysis	Raw *p*	Bonferroni P	BH *q*‐value (FDR)	Holm P	Significance status
Multiple testing corrections					
NAION all‐time	0.011	0.088	0.015	0.033	FDR/Holm significant
NAION 3‐year	0.014	0.112	0.016	0.028	FDR/Holm significant
Optic atrophy all‐time	< 0.001	< 0.001	< 0.001	< 0.001	Robust (all corrections)
Optic atrophy 5‐year	< 0.001	< 0.001	< 0.001	< 0.001	Robust (all corrections)
Optic atrophy 3‐year	< 0.001	< 0.001	< 0.001	< 0.001	Robust (all corrections)
Optic atrophy 1‐year	< 0.001	0.003	< 0.001	0.002	Robust (all corrections)
Optic atrophy diabetic	0.045	0.360	0.045	0.045	FDR/Holm significant
Optic atrophy non‐diabetic	< 0.001	< 0.001	< 0.001	< 0.001	Robust (all corrections)
Test method	Statistic	*p*	OR estimate	—	—
Alternative statistical tests (NAION all‐time)					
Fisher's exact test	—	0.017	0.428	—	—
Mid‐P test	—	0.014	—	—	—
Chi‐square (yates correction)	χ^2^ = 5.63	0.018	—	—	—
Chi‐square (uncorrected)	χ^2^ = 6.41	0.011	—	—	—
Z‐test for proportions	Z = −2.53	0.011	—	—	—
Estimation method	NAION OR	95% CI	Interpretation	—	—
Penalized and corrected estimates (NAION)					
Maximum likelihood (standard)	0.428	0.218–0.842	Unadjusted estimate	—	—
Firth‐corrected (penalized)	0.438	—	Reduced small‐sample bias	—	—
Empirical Bayes shrinkage	0.563	—	Shrunk toward null	—	—
Bootstrap median (10,000 replicates)	0.424	0.192–0.818	Percentile CI	—	—
Bootstrap *p* (OR < 1)	99.4%	—	Probability of protection	—	—

Abbreviations: BH, Benjamini‐Hochberg; CI, confidence interval; FDR, false discovery rate; NAION, non‐arteritic anterior ischemic optic neuropathy; OR, odds ratio.

**FIGURE 5:**
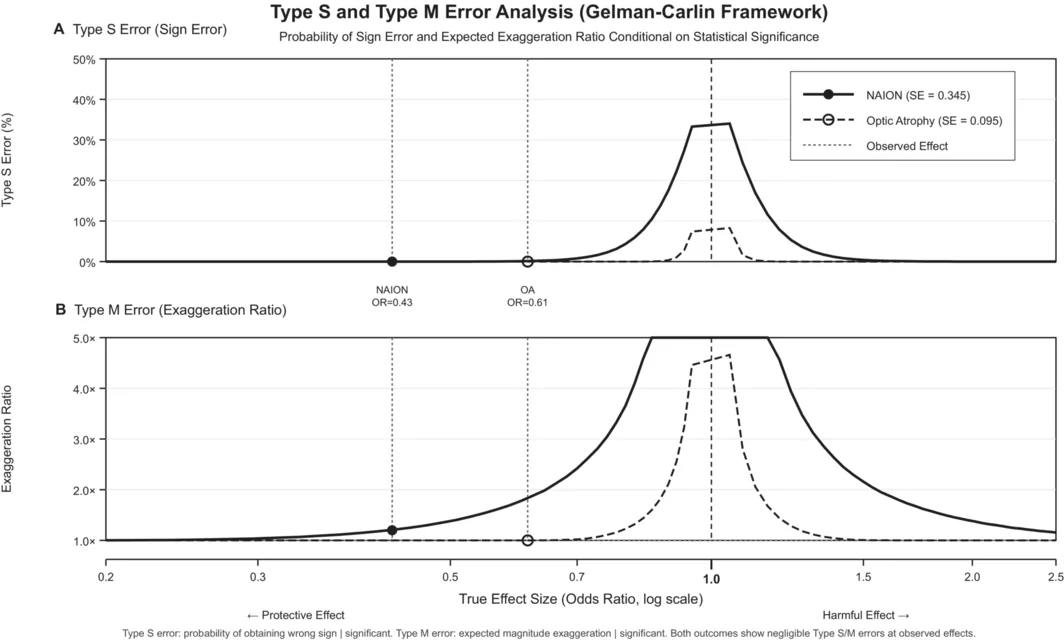
Type S and type M error estimation plot.

### 3.6 Supplementary Sensitivity Analyses

Leave‐one‐out sensitivity analysis (Table S2) demonstrated that NAION findings would lose significance with the addition of three events to the GLP‐1 RA group, while optic atrophy findings remained significant even with the addition of 66 events. Probabilistic bias analysis (Table S3) demonstrated that after accounting for residual BMI confounding and simulated unmeasured confounding, the probability of a protective effect remained 99.7% for NAION and 100% for optic atrophy. Replication probability simulations (Table S4) demonstrated 69.3% replication probability for NAION and 99.9% for optic atrophy at the current sample size.

## 4 Discussion

This study evaluated the relationship between GLP‐1 RA use and optic outcomes in IIH patients using a large propensity score‐matched cohort from the TriNetX Global Collaborative Network. It found that GLP‐1 RA use demonstrated significant protective associations against NAION and optic atrophy, with protective associations observed across multiple time windows and subgroup analyses. Importantly, these findings in IIH patients should not be directly compared to reports of increased NAION risk with GLP‐1 RAs in general populations, as IIH patients represent a fundamentally different clinical context in which papilledema is a defining feature. The weight reduction achieved through GLP‐1 RA therapy may alleviate papilledema and reduce the risk of secondary optic nerve damage, a mechanism absent in the general population.

Our findings are consistent with recent systematic reviews demonstrating that GLP‐1 RAs reduce papilledema risk by 53%–75% and visual disturbances by 59%–72% in IIH patients [27, 28]. While these studies focused on broader IIH outcomes, our study uniquely examines optic nerve complications specifically. Further, the seminal study by Hathaway et al. [25] reported significantly increased NAION risk with semaglutide among individuals with type 2 diabetes (HR 4.28, 95% CI 1.62–11.29) and among individuals who were overweight or obese (HR 7.64, 95% CI 2.21–26.36). However, this single‐center study included only 46 NAION cases across all groups, raising concerns about statistical precision. In addition, a Danish‐Norwegian cohort study by Simonsen et al. has demonstrated an elevated NAION risk associated with semaglutide use, with a pooled HR of 2.81 (95% CI 1.67–4.75) compared to sodium‐glucose co‐transporter two inhibitors [32]. In contrast, a TriNetX‐based study by Chou et al. found no significant correlation between semaglutide and NAION among individuals with T2DM, obesity, or both conditions [33]. Likewise, a large multicenter study by Cai et al. utilizing the OHDSI network across 14 databases with 37.1 million individuals has demonstrated no significant difference in NAION risk between semaglutide users and those prescribed other second‐line antidiabetic medications using an active‐comparator cohort design. However, a modest increase was observed in self‐controlled case‐series analysis (incidence rate ratio 1.32, 95% CI 1.14–1.54) [34].

Importantly, the observed protective association in IIH patients is not necessarily divergent from general population findings, but rather reflects the distinct pathophysiology of IIH. IIH patients characteristically have papilledema, which is part of the diagnostic criteria and is itself a recognized risk factor for NAION. When GLP‐1 RAs induce weight loss in these patients, the resulting reduction in intracranial pressure leads to papilledema alleviation, thereby reducing the mechanical and vascular stress on the optic nerve. This mechanism is entirely absent in general population studies where papilledema is not present. Therefore, the populations are fundamentally non‐comparable; this study specifically evaluated IIH patients who carry distinct pathophysiological characteristics compared to general diabetic or obese populations [35, 36, 37, 38, 39]. First, IIH patients have elevated intracranial pressure that can compromise optic nerve perfusion through papilledema, and weight reduction achieved through GLP‐1 RA therapy has been proposed to reduce intracranial pressure, alleviate papilledema, and improve optic nerve perfusion [40]. Second, methodological differences, including matching variables, follow‐up duration, and outcome ascertainment, may contribute to heterogeneity across studies. Third, the baseline risk profile differs significantly; IIH patients already carry elevated risk for optic complications due to papilledema, and the potential benefits from papilledema reduction may outweigh possible adverse vascular effects in this population, though this remains to be tested directly [41].

In this study, the stronger protective effect observed in non‐diabetic IIH patients (47.5% relative risk reduction) compared to diabetic patients (24.4% relative risk reduction) provides mechanistic insights. This pattern suggests that the protective association may be mediated primarily by weight‐reduction effects rather than confounded by the indication for diabetes treatment. In non‐diabetic patients where diabetes‐related confounding is absent, the weight loss benefits of GLP‐1 RAs on intracranial pressure reduction and papilledema alleviation may be more pronounced. This interpretation aligns with the hypothesis that weight reduction represents a primary mechanism of benefit in IIH, as excess body weight has been strongly linked to elevated intracranial pressure and worse visual outcomes. Notably, the reduced optic atrophy observed in the GLP‐1 RA cohort is most likely attributable to greater weight reduction leading to papilledema resolution and IIH remission, rather than a direct neuroprotective effect of GLP‐1 RAs on the optic nerve. This distinction is clinically important: the benefit derives from the downstream consequence of weight loss on intracranial pressure dynamics, not from an intrinsic pharmacological protection of the optic nerve by GLP‐1 RAs.

In June 2025, the European Medicines Agency Pharmacovigilance Risk Assessment Committee concluded that NAION represents an uncommon adverse event associated with semaglutide, possibly affecting up to one in 10,000 users, and recommended updating product information accordingly [42, 43]. The World Health Organization subsequently issued a safety alert regarding this association. These regulatory actions reflect the uncertainty in the current evidence base and the need for continued pharmacovigilance [42, 43]. The current study findings suggest that IIH patients may represent a population in which the benefit–risk profile differs from that of the general population, as GLP‐1 RA‐induced weight reduction may alleviate papilledema and thereby indirectly reduce the risk of optic nerve complications in this specific population.

Residual BMI imbalance after matching (SMD = 0.642) is the central unresolved concern in this study. The probabilistic bias analysis is informative, but it does not rule out BMI confounding as an explanation for what we observed. The GLP‐1 RA cohort had higher baseline BMI (41.6 versus 35.6 kg/m^2^), which could bias against detecting protective effects, as higher BMI is associated with worse IIH outcomes. Probabilistic bias analysis demonstrated that after accounting for this residual confounding, the probability of an actual protective effect remained 99.7% for NAION and 100% for optic atrophy. The E‐values of 4.09 for NAION and 2.62 for optic atrophy indicate that an unmeasured confounder would need significant association with both GLP‐1 RA use and optic outcomes to fully explain the observed effects. We acknowledge that direct adjustment for BMI as a covariate in the post‐matching Cox regression would have been preferable. However, the TriNetX platform's built‐in Cox regression function does not support the inclusion of additional covariates beyond the propensity score‐matched design. Importantly, the direction of BMI confounding bias operates against our findings, as the GLP‐1 RA cohort had higher baseline BMI, which is associated with worse IIH outcomes and higher risk of optic complications, yet this group demonstrated lower event rates.

Several limitations warrant recognition in this study. First, this study relied on ICD‐10 diagnostic codes for case ascertainment, which carries inherent misclassification risk. The G93.2 code for IIH has a positive predictive value (PPV) of 63%, increasing to 82% when assigned by ophthalmologists [44]. The H47.01 code for NAION has a PPV of 74.5%, improving to 86.8% when patients are evaluated by neuro‐ ophthalmologists [45]. However, if misclassification was non‐differential between exposure groups, this would bias results conservatively toward the null. Our methodology aligns with recently published TriNetX studies examining GLP‐1 Ras in IIH [28, 31]. Nevertheless, prospective validation with medical record review would strengthen confidence in these findings. Our cohort was likely enriched for true cases given the specialty care settings and strict exclusion criteria, and the consistent protective pattern observed across two different optic outcomes supports the validity of these associations.

Second, the retrospective observational approach cannot establish causality, and residual confounding remains possible despite PSM. Third, the GLP‐1 RA cohort had substantially shorter follow‐up (747 versus 1239 days), which means a narrower window to observe events in the exposed group. That alone could produce lower event counts regardless of any true difference. HR was used as the primary effect measure to address these concerns, and time‐stratified analyses were consistent, but the unequal follow‐up time is not an issue that statistical adjustment alone can fully correct. Fourth, this study defined optic atrophy broadly since the TriNetX platform does not provide specific coding for post‐NAION optic atrophy, and the authors acknowledge this may have captured heterogeneous etiologies. Fifth, the fragility index of three for NAION indicates that small changes in event counts could alter statistical significance, warranting cautious interpretation and replication in larger cohorts. Sixth, the NAION finding remained significant after FDR correction but not after Bonferroni correction, though the optic atrophy findings remained significant across all correction methods. Seventh, the Cox proportional hazards model employed by the TriNetX platform did not account for the matched structure of the cohort through stratification by matched pairs or robust sandwich variance estimators, which may result in underestimated standard errors. However, the consistency of results across multiple independent analytical approaches, including risk ratios, odds ratios, hazard ratios, Fisher's exact test, Firth‐corrected estimates, bootstrap analysis, and RMST analysis, supports the robustness of the findings despite this methodological limitation.

Despite these limitations, this study possesses multiple strengths. The substantial cohort size with over 31,000 matched patients provided adequate statistical power. The use of multiple analytical approaches, including time‐stratified analyses, subgroup analyses, multiple testing corrections, and probabilistic bias analysis, strengthened the validity of the study outcomes. The replication probability of 99.9% for optic atrophy suggests a high likelihood of replication in future studies.

Moreover, the present study findings suggest that GLP‐1 RA use among IIH patients is associated with a reduced risk of NAION and optic atrophy. These results should not be directly compared to reports of increased NAION risk in general populations, as IIH patients represent a distinct clinical population in which papilledema is present at baseline and weight reduction through GLP‐1 RA therapy may alleviate papilledema and thereby reduce optic nerve complications. Prospective investigations are needed to validate these findings and examine the pathophysiological mechanisms underlying the observed protective associations.

## 5 Conclusions

This study highlighted that GLP‐1 RA use among IIH patients was associated with a significantly decreased risk of NAION and optic atrophy. The protective association was observed across multiple time windows and remained significant after multiple testing corrections for optic atrophy outcomes. The NAION finding was based on 12 events in the exposed cohort with a fragility index of 3, indicating that statistical significance would be lost with minimal changes in event counts; this result warrants cautious interpretation. The greater protective effect in non‐diabetic patients suggests that weight‐reduction mechanisms, particularly papilledema alleviation through intracranial pressure reduction, likely mediate the observed associations. These findings apply specifically to IIH patients in whom papilledema is present at baseline and should not be extrapolated to infer optic neuroprotection in general populations where recent pharmacovigilance signals have suggested increased NAION risk with GLP‐1 RAs. The observed protective association is best explained by the weight‐reducing and papilledema‐alleviating properties of GLP‐1 RAs in IIH rather than a direct neuroprotective effect. However, given the retrospective observational design, residual confounding cannot be excluded. Prospective studies and randomized controlled trials are needed to test these associations and determine whether GLP‐1 RAs have a role in reducing optic complications in IIH.

## Author Contributions

**Faisal A. Al‐Harbi:** conceptualization, methodology, investigation, data curation, writing – original draft. **Mohanad A. Alkuwaiti:** conceptualization, methodology, investigation, data curation, writing – original draft. **Yazeed B. Alaql:** investigation, data curation, validation, writing – original draft. **Ahmed K. Alsaif:** investigation, data curation, validation, writing – original draft. **Ahmed A. Alessa:** investigation, data curation, visualization, writing – original draft. **Meshari Ayed Alharbi:** investigation, validation, writing – original draft. **Mohammed Alfalah:** investigation, resources, writing – review and editing. **Saud A. Alnaaim:** investigation, resources, writing – review and editing. **Sajjad M. AlHaddad:** resources, validation, writing – review and editing. **Ahmed Y. Azzam:** conceptualization, methodology, software, formal analysis, validation, resources, data curation, writing – review and editing, visualization, supervision, project administration.

## Funding

The authors have nothing to report.

## Conflicts of Interest

The authors declare no conflicts of interest.

## Supporting information


**Figure S1:** Love plot for covariate balance before and after propensity score matching.

**Figure S2:** Multiple testing correction plot.

**Table S1:** ICD‐10 diagnostic codes and medication classifications.

**Table S2:** Leave‐one‐out sensitivity analysis.

**Table S3:** Probabilistic bias analysis (monte carlo simulation).

**Table S4:** Replication probability simulations.

## Data Availability

The data that support the findings of this study are available from TriNetX. Restrictions apply to the availability of these data, which were used under license for this study. Data are available from the author(s) with the permission of TriNetX.
