# Supplementary material for: Risk of Non‐Arteritic Anterior Ischemic Optic Neuropathy in Idiopathic Intracranial Hypertension Patients Treated with GLP‐1 Receptor Agonists
Source: Ann Clin Transl Neurol. 2026 Apr 17;13(11):2289–302. doi: 10.1002/acn3.70406 (PMC13395034; doi:10.1002/acn3.70406)
Supplement: Supplementary file 2 [file ACN3-13-2289-s003.pdf]

Multiple Testing Correction

Comparison of Bonferroni, Benjamini-Hochberg FDR (Q-values), and Holm Step-Down Corrections (8 Tests, α = 0.05)

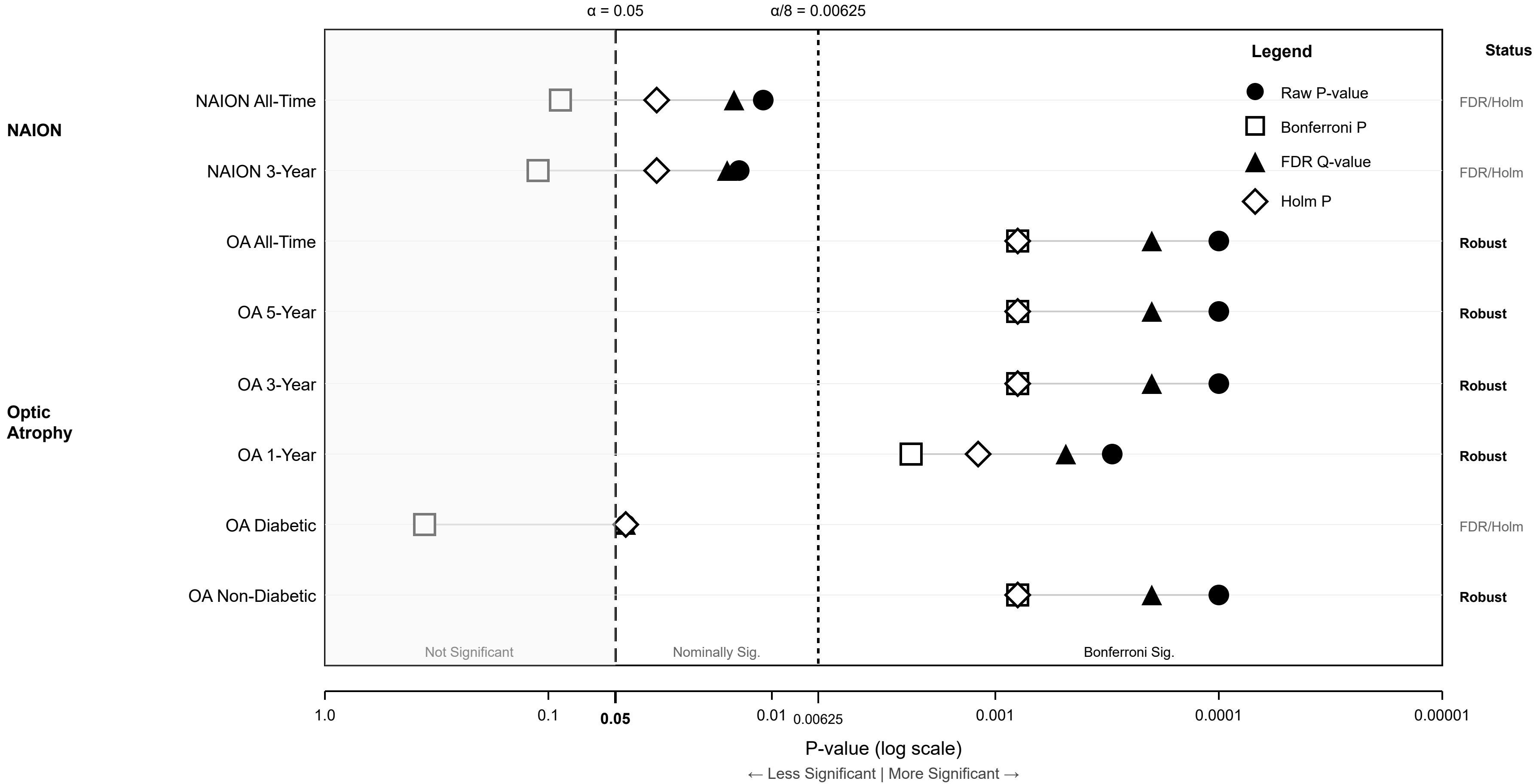

8 hypothesis tests with Bonferroni threshold α/8 = 0.00625. "Robust" = significant after all corrections; "FDR/Holm" = significant after FDR and Holm but not Bonferroni.
