# Supplementary material for: Risk of Non‐Arteritic Anterior Ischemic Optic Neuropathy in Idiopathic Intracranial Hypertension Patients Treated with GLP‐1 Receptor Agonists
Source: Ann Clin Transl Neurol. 2026 Apr 17;13(11):2289–302. doi: 10.1002/acn3.70406 (PMC13395034; doi:10.1002/acn3.70406)
Supplement: Supplementary file 3 [file ACN3-13-2289-s001.docx]

**Supplementary Table 1:** ICD-10 Diagnostic Codes and Medication Classifications.

| **Category** | **Code/Classification** | **Description** |
| --- | --- | --- |
| **Cohort Definition** | | |
| Idiopathic intracranial hypertension | G93.2 | Benign intracranial hypertension (idiopathic intracranial hypertension) |
| **Exposure: GLP-1 Receptor Agonists** | | |
| Semaglutide | ATC A10BJ06 | Includes Ozempic®, Wegovy®, Rybelsus® |
| Liraglutide | ATC A10BJ02 | Includes Victoza®, Saxenda® |
| Dulaglutide | ATC A10BJ05 | Includes Trulicity® |
| Exenatide | ATC A10BJ01 | Includes Byetta®, Bydureon® |
| Tirzepatide | ATC A10BX16 | Includes Mounjaro®, Zepbound® |
| Lixisenatide | ATC A10BJ03 | Includes Adlyxin® |
| **Primary Outcome** | | |
| Non-arteritic anterior ischemic optic neuropathy | H47.01 | Ischemic optic neuropathy |
| **Secondary Outcome** | | |
| Optic atrophy, unspecified | H47.20 | Optic atrophy, unspecified eye |
| Primary optic atrophy | H47.21 | Primary optic atrophy (all laterality codes) |
| Hereditary optic atrophy | H47.22 | Leber's optic atrophy |
| Other optic atrophy | H47.23 | Other optic atrophy (all laterality codes) |
| **Comorbidities** | | |
| Diabetes mellitus | E08–E13 | All diabetes mellitus diagnoses |
| Diabetes due to underlying condition | E08 | Diabetes mellitus due to underlying condition |
| Drug/chemical-induced diabetes | E09 | Drug or chemical induced diabetes mellitus |
| Type 1 diabetes mellitus | E10 | Type 1 diabetes mellitus |
| Type 2 diabetes mellitus | E11 | Type 2 diabetes mellitus |
| Other specified diabetes mellitus | E13 | Other specified diabetes mellitus |
| Disorders of lipoprotein metabolism | E78 | Disorders of lipoprotein metabolism and other lipidemias |
| Pure hypercholesterolemia | E78.0 | Pure hypercholesterolemia, including familial |
| Pure hyperglyceridemia | E78.1 | Pure hyperglyceridemia |
| Mixed hyperlipidemia | E78.2 | Mixed hyperlipidemia |
| Hyperlipidemia, unspecified | E78.5 | Hyperlipidemia, unspecified |
| Diseases of the circulatory system | I00–I99 | ICD-10-CM Chapter 9: Diseases of the circulatory system |
| Diseases of the eye and adnexa | H00–H59 | ICD-10-CM Chapter 7: Diseases of the eye and adnexa |
| **Propensity Score Matching Variables** | | |
| Central nervous system agents | — | Therapeutic class: CNS medications |
| Ophthalmic agents | — | Therapeutic class: Ophthalmic preparations |
| **Clinical Measurements** | | |
| Body mass index | — | Recorded BMI values (kg/m²) |
| Systolic blood pressure | — | Recorded systolic BP (mmHg) |
| Diastolic blood pressure | — | Recorded diastolic BP (mmHg) |
| Heart rate | — | Recorded heart rate (beats per minute) |

***Notes:*** *ICD-10-CM codes based on the International Classification of Diseases, Tenth Revision, Clinical Modification (2024). ATC codes based on the World Health Organization Anatomical Therapeutic Chemical Classification System. TriNetX utilizes standardized terminologies including ICD-10-CM for diagnoses and RxNorm for medications; specific RxCUI codes for individual GLP-1 receptor agonist formulations are mapped to the ATC classifications listed above. Diabetes mellitus codes E08–E13 were explicitly specified in the cohort definition and confirmed in the baseline characteristics. Diseases of the circulatory system (I00–I99) and diseases of the eye and adnexa (H00–H59) represent chapter-level classifications encompassing all diagnoses within those ICD-10-CM chapters. Propensity score matching additionally included demographic variables (age, sex, race, ethnicity) not shown in this table. — indicates medication therapeutic class without specific ICD-10 code.* ***Abbreviations:*** *ATC, Anatomical Therapeutic Chemical; BMI, body mass index; BP, blood pressure; CNS, central nervous system; GLP-1, glucagon-like peptide-1; ICD-10-CM, International Classification of Diseases, Tenth Revision, Clinical Modification; RxCUI, RxNorm Concept Unique Identifier.*
