# Supplementary material for: Risk of Non‐Arteritic Anterior Ischemic Optic Neuropathy in Idiopathic Intracranial Hypertension Patients Treated with GLP‐1 Receptor Agonists
Source: Ann Clin Transl Neurol. 2026 Apr 17;13(11):2289–302. doi: 10.1002/acn3.70406 (PMC13395034; doi:10.1002/acn3.70406)
Supplement: Supplementary file 4 [file ACN3-13-2289-s004.docx]

**Supplementary Table 2:** Leave-One-Out Sensitivity Analysis.

| **Scenario** | **GLP-1 RA Events** | **No GLP-1 RA Events** | **Odds Ratio** | **95% CI** | **P-value** | **Significant** |
| --- | --- | --- | --- | --- | --- | --- |
| **NAION — Removing Events from GLP-1 RA Group** | | | | | | |
| Base case | 12 | 28 | 0.428 | 0.218–0.842 | 0.017 | Yes |
| Remove 1 event | 11 | 28 | 0.392 | 0.195–0.789 | 0.009 | Yes |
| Remove 2 events | 10 | 28 | 0.357 | 0.173–0.735 | 0.005 | Yes |
| Remove 3 events | 9 | 28 | 0.321 | 0.151–0.681 | 0.003 | Yes |
| Remove 4 events | 8 | 28 | 0.285 | 0.130–0.626 | 0.001 | Yes |
| Remove 5 events | 7 | 28 | 0.250 | 0.109–0.572 | <0.001 | Yes |
| **NAION — Removing Events from No GLP-1 RA Group** | | | | | | |
| Base case | 12 | 28 | 0.428 | 0.218–0.842 | 0.017 | Yes |
| Remove 1 event | 12 | 27 | 0.444 | 0.225–0.877 | 0.024 | Yes |
| Remove 2 events | 12 | 26 | 0.461 | 0.233–0.914 | 0.033 | Yes |
| Remove 3 events | 12 | 25 | 0.480 | 0.241–0.955 | 0.047 | Yes |
| Remove 4 events† | 12 | 24 | 0.500 | 0.250–0.999 | 0.065 | No |
| Remove 5 events | 12 | 23 | 0.521 | 0.259–1.048 | 0.089 | No |
| **NAION — Adding Events to GLP-1 RA Group (Fragility Assessment)** | | | | | | |
| Base case | 12 | 28 | 0.428 | 0.218–0.842 | 0.017 | Yes |
| Add 1 event | 13 | 28 | 0.464 | 0.240–0.896 | 0.027 | Yes |
| Add 2 events | 14 | 28 | 0.500 | 0.263–0.949 | 0.043 | Yes |
| Add 3 events† | 15 | 28 | 0.535 | 0.286–1.003 | 0.066 | No |
| Add 4 events | 16 | 28 | 0.571 | 0.309–1.056 | 0.096 | No |
| Add 5 events | 17 | 28 | 0.607 | 0.332–1.109 | 0.135 | No |
| **Optic Atrophy — Selected Scenarios** | | | | | | |
| Base case | 179 | 295 | 0.613 | 0.508–0.739 | <0.001 | Yes |
| Remove 5 from GLP-1 RA | 174 | 295 | 0.596 | 0.493–0.720 | <0.001 | Yes |
| Remove 10 from GLP-1 RA | 169 | 295 | 0.578 | 0.477–0.701 | <0.001 | Yes |
| Add 10 to GLP-1 RA | 189 | 295 | 0.648 | 0.539–0.779 | <0.001 | Yes |
| Add 20 to GLP-1 RA | 199 | 295 | 0.682 | 0.569–0.818 | <0.001 | Yes |
| Add 66 to GLP-1 RA† | 245 | 295 | 0.844 | 0.712–1.001 | 0.051 | No |
| **Summary of Fragility Analysis** | **NAION** | **Optic Atrophy** | — | — | — | — |
| Fragility Index (adding to GLP-1 RA) | 3 | 66 | — | — | — | — |
| Fragility Index (removing from No GLP-1 RA) | 4 | 74 | — | — | — | — |
| Minimum Fragility Index | 3 | 66 | — | — | — | — |
| Interpretation | Fragile | Robust | — | — | — | — |
| OR range (removing 1–3 from GLP-1 RA) | 0.321–0.392 | — | — | — | — | — |
| OR range (removing 1–3 from No GLP-1 RA) | 0.444–0.480 | — | — | — | — | — |

***Notes:*** *†Indicates the scenario at which statistical significance is lost (P > 0.05). Leave-one-out sensitivity analysis systematically removes or adds events to assess the stability of findings. The Fragility Index represents the minimum number of patients whose event status would need to change to reverse statistical significance at α = 0.05. For NAION, the Fragility Index of 3 indicates that adding just 3 events to the GLP-1 RA group (or removing 4 from the No GLP-1 RA group) would render the finding non-significant; this fragility warrants cautious interpretation and replication in larger cohorts. For optic atrophy, the Fragility Index of 66 indicates highly robust findings that would require substantial changes in event counts to lose significance. P-values calculated using Fisher's exact test. All scenarios maintain original denominators (NAION: 15,559 and 15,560; Optic Atrophy: 15,029 and 15,298 for GLP-1 RA and No GLP-1 RA groups, respectively).* ***Abbreviations:*** *CI, confidence interval; GLP-1 RA, glucagon-like peptide-1 receptor agonist; NAION, non-arteritic anterior ischemic optic neuropathy; OR, odds ratio.*
