# Supplementary material for: Risk of Non‐Arteritic Anterior Ischemic Optic Neuropathy in Idiopathic Intracranial Hypertension Patients Treated with GLP‐1 Receptor Agonists
Source: Ann Clin Transl Neurol. 2026 Apr 17;13(11):2289–302. doi: 10.1002/acn3.70406 (PMC13395034; doi:10.1002/acn3.70406)
Supplement: Supplementary file 5 [file ACN3-13-2289-s005.docx]

**Supplementary Table 4:** Replication Probability Simulations.

| **Parameter/Metric** | **NAION** | **Optic Atrophy** |
| --- | --- | --- |
| **Current Study Characteristics** | | |
| Observed Odds Ratio | 0.428 | 0.613 |
| Log(OR) | −0.849 | −0.489 |
| Standard Error of Log(OR) | 0.345 | 0.095 |
| Total Sample Size | 31,119 | 30,327 |
| Statistical Power (at observed effect) | 69.1% | 99.9% |
| **Replication Probability at Same Sample Size (N = 10,000 simulations)** | | |
| Assuming true effect equals observed effect |  |  |
| Replication probability (same direction, P < 0.05) | 69.3% | 99.9% |
| Probability of any significant result | 69.3% | 99.9% |
| Probability of non-significant result | 30.7% | 0.1% |
| Probability of opposite direction (Type S error) | 0.0% | 0.0% |
| Expected median OR in replication | 0.428 | 0.613 |
| Expected mean OR in replication | 0.454 | 0.617 |
| **Power for Replication at Various True Effect Sizes** | | |
| True OR = 0.30 | 93.7% | — |
| True OR = 0.40 | 75.6% | — |
| True OR = 0.428 (observed NAION) | 69.1% | — |
| True OR = 0.50 | 51.9% | 100.0% |
| True OR = 0.55 | — | 100.0% |
| True OR = 0.60 | 31.6% | 100.0% |
| True OR = 0.613 (observed OA) | — | 99.9% |
| True OR = 0.70 | 17.8% | 96.2% |
| True OR = 0.80 | 9.9% | 64.7% |
| True OR = 0.90 | — | 19.7% |
| True OR = 1.00 (null) | 5.0% | 5.0% |
| **Sample Size Requirements for Adequate Power** | | |
| Current sample size | 31,119 | 30,327 |
| N required for 80% power | 40,418 | Already achieved |
| N required for 90% power | 54,109 | Already achieved |
| Sample size multiplier for 80% power | 1.30× | 0.30× |
| Sample size multiplier for 90% power | 1.74× | 0.40× |
| **Replication Probability by Study Size** | | |
| *Half current size (N ≈ 15,500)* |  |  |
| Standard Error | 0.488 | 0.135 |
| Power | 41.2% | 95.2% |
| Replication probability | 41.5% | 95.0% |
| *Current size (N ≈ 31,000)* |  |  |
| Standard Error | 0.345 | 0.095 |
| Power | 69.1% | 99.9% |
| Replication probability | 69.3% | 99.9% |
| *1.5× current size (N ≈ 46,500)* |  |  |
| Standard Error | 0.282 | 0.078 |
| Power | 85.3% | 100.0% |
| Replication probability | 85.2% | 100.0% |
| *2× current size (N ≈ 62,000)* |  |  |
| Standard Error | 0.244 | 0.067 |
| Power | 93.5% | 100.0% |
| Replication probability | 93.4% | 100.0% |
| *3× current size (N ≈ 93,000)* |  |  |
| Standard Error | 0.199 | 0.055 |
| Power | 98.9% | 100.0% |
| Replication probability | 98.9% | 100.0% |
| **Expected Effect Size Distribution in Replications** | | |
| Assuming true OR equals observed OR |  |  |
| Expected median OR | 0.428 | 0.613 |
| Expected mean OR | 0.454 | 0.616 |
| 50% prediction interval (IQR) | 0.339–0.538 | 0.575–0.654 |
| 90% prediction interval | 0.245–0.757 | 0.523–0.717 |
| **Replication Under Different True Effect Assumptions** | | |
| *Assumed true OR = 0.30 (NAION only)* |  |  |
| Replication probability | 93.8% | — |
| Type S error probability | 0.0% | — |
| Interpretation | Likely to replicate | — |
| *Assumed true OR = 0.50* |  |  |
| Replication probability | 51.3% | 100.0% |
| Type S error probability | 0.0% | 0.0% |
| Interpretation | May/may not replicate | Likely to replicate |
| *Assumed true OR = 0.60* |  |  |
| Replication probability | 31.9% | 100.0% |
| Type S error probability | 0.0% | 0.0% |
| Interpretation | Unlikely to replicate | Likely to replicate |
| *Assumed true OR = 0.80* |  |  |
| Replication probability | 9.6% | 64.7% |
| Type S error probability | 0.5% | 0.0% |
| Interpretation | Unlikely to replicate | May/may not replicate |
| *Assumed true OR = 1.00 (null effect)* |  |  |
| Replication probability | 2.6% | 2.5% |
| Type S error probability | 2.5% | 2.8% |
| Interpretation | False positive rate | False positive rate |

***Notes:*** *Replication probability simulations performed using Monte Carlo methods with 10,000 iterations per scenario. For each simulation, observed log(OR) values were drawn from a normal distribution with mean equal to the assumed true log(OR) and standard deviation equal to the standard error of the log(OR) estimate. Replication was defined as achieving statistical significance (two-sided P < 0.05) in the same direction as the original finding. Power calculations assume equal group sizes and the same event rates as the original study. Type S error represents the probability of obtaining a statistically significant result in the opposite direction of the true effect. The 50% and 90% prediction intervals represent the range of OR values expected in 50% and 90% of replication studies, respectively, assuming the observed effect is the true effect. For NAION, the current study has moderate power (69.1%) with ~31% chance of a non-significant result even if the true effect equals the observed effect; a sample size of approximately 40,000 would be needed for 80% power. For optic atrophy, the current study has excellent power (99.9%) with near-certain replication expected. — indicates scenario not applicable or not calculated for that outcome.* ***Abbreviations:*** *IQR, interquartile range; N, sample size; NAION, non-arteritic anterior ischemic optic neuropathy; OA, optic atrophy; OR, odds ratio; SE, standard error.*
