# Supplementary material for: Risk of Non‐Arteritic Anterior Ischemic Optic Neuropathy in Idiopathic Intracranial Hypertension Patients Treated with GLP‐1 Receptor Agonists
Source: Ann Clin Transl Neurol. 2026 Apr 17;13(11):2289–302. doi: 10.1002/acn3.70406 (PMC13395034; doi:10.1002/acn3.70406)
Supplement: Supplementary file 6 [file ACN3-13-2289-s006.docx]

**Supplementary Table 3:** Probabilistic Bias Analysis (Monte Carlo Simulation).

| **Parameter/Metric** | **NAION** | **Optic Atrophy** |
| --- | --- | --- |
| **Observed Effect Estimates** | | |
| Observed Odds Ratio | 0.428 | 0.613 |
| 95% Confidence Interval | 0.218–0.842 | 0.508–0.739 |
| Log(OR) | −0.849 | −0.489 |
| Standard Error of Log(OR) | 0.345 | 0.095 |
| **Bias Parameters and Distributions** | | |
| *BMI Confounding* |  |  |
| Observed BMI difference (GLP-1 − No GLP-1), kg/m² | 6.0 ± 1.0 | 6.0 ± 1.0 |
| OR per 5 kg/m² BMI increase (literature-based) | 1.15 ± 0.05 | 1.15 ± 0.05 |
| Bias direction | Against protection | Against protection |
| *Unmeasured Confounding* |  |  |
| Distribution | Triangular (0.8, 1.0, 1.3) | Triangular (0.8, 1.0, 1.3) |
| Bias direction | Uncertain | Uncertain |
| *Selection Bias* |  |  |
| Distribution | Triangular (0.9, 1.0, 1.1) | Triangular (0.9, 1.0, 1.1) |
| Bias direction | Uncertain | Uncertain |
| **Monte Carlo Simulation Results (N = 10,000)** | | |
| Bias-corrected OR, mean | 0.399 | 0.540 |
| Bias-corrected OR, median | 0.373 | 0.534 |
| Bias-corrected OR, SD | 0.152 | 0.084 |
| 95% Simulation Interval | 0.181–0.779 | 0.394–0.723 |
| Probability OR < 1.0 (protective) | 99.7% | 100.0% |
| Probability OR < 0.8 (meaningfully protective) | 98.0% | 99.6% |
| Probability OR > 1.0 (harmful) | 0.4% | 0.0% |
| **Deterministic Sensitivity Scenarios** | | |
| *Scenario 1: Base (observed)* |  |  |
| Corrected OR | 0.428 | 0.613 |
| Interpretation | Strongly protective | Moderately protective |
| *Scenario 2: BMI correction only* |  |  |
| Corrected OR | 0.363 | 0.519 |
| Interpretation | Strongly protective | Strongly protective |
| *Scenario 3: Moderate unmeasured confounding (×1.3)* |  |  |
| Corrected OR | 0.556 | 0.797 |
| Interpretation | Moderately protective | Moderately protective |
| *Scenario 4: Strong unmeasured confounding (×1.5)* |  |  |
| Corrected OR | 0.642 | 0.919 |
| Interpretation | Moderately protective | Weakly protective |
| *Scenario 5: BMI + moderate unmeasured* |  |  |
| Corrected OR | 0.472 | 0.675 |
| Interpretation | Strongly protective | Moderately protective |
| *Scenario 6: BMI + strong unmeasured* |  |  |
| Corrected OR | 0.544 | 0.779 |
| Interpretation | Moderately protective | Moderately protective |
| *Scenario 7: Extreme scenario (×2.0, unlikely)* |  |  |
| Corrected OR | 0.725 | 1.039 |
| Interpretation | Moderately protective | Near null |
| **Bias Factor Required to Nullify Effect** | | |
| Observed OR | 0.428 | 0.613 |
| After BMI correction (OR/1.18) | 0.363 | 0.519 |
| Bias factor needed to nullify | 2.76 | 1.92 |
| Corresponding unmeasured confounder RR | ≥2.8 | ≥1.9 |
| E-value (point estimate) | 4.09 | 2.62 |
| E-value (confidence interval bound) | 1.66 | 2.03 |
| Interpretation | Very robust | Moderately robust |

***Notes:*** *Monte Carlo simulation performed with 10,000 iterations using the following approach: (1) sample bias parameters from specified distributions; (2) calculate bias-corrected log(OR) = observed log(OR) − log(BMI bias factor) + log(unmeasured confounding) + log(selection bias) + random error; (3) exponentiate to obtain bias-corrected OR; (4) summarize distribution of corrected estimates. BMI confounding: GLP-1 RA group had 6.0 kg/m² higher mean BMI after propensity score matching (41.6 vs 35.6 kg/m²); since higher BMI is associated with worse intracranial hypertension outcomes, this creates bias AGAINST finding a protective effect, meaning the observed protective association is likely CONSERVATIVE (underestimated). The bias factor for BMI confounding was calculated as OR^(BMI difference/5) where OR per 5 kg/m² was estimated at 1.15 based on IIH literature. Unmeasured confounding was modeled using a triangular distribution with mode 1.0 (no bias) and range 0.8–1.3, representing modest residual confounding scenarios. Selection bias was modeled similarly with range 0.9–1.1. Deterministic scenarios show bias-corrected ORs under specific assumptions; BMI correction alone makes the protective effect STRONGER (ORs 0.363 and 0.519), while unmeasured confounding scenarios attenuate the effect toward the null. Even under extreme assumptions (unmeasured confounding factor of 2.0), NAION remains protective (OR 0.725) and optic atrophy reaches the null (OR 1.039). The E-value represents the minimum strength of association an unmeasured confounder would need with both treatment and outcome to fully explain the observed effect; values >2 suggest robust findings.* ***Abbreviations:*** *BMI, body mass index; CI, confidence interval; E-value, evidence value for unmeasured confounding; GLP-1 RA, glucagon-like peptide-1 receptor agonist; IIH, idiopathic intracranial hypertension; NAION, non-arteritic anterior ischemic optic neuropathy; OR, odds ratio; RR, risk ratio; SD, standard deviation.*
