# Supplementary material for: Risk of Non‐Arteritic Anterior Ischemic Optic Neuropathy in Idiopathic Intracranial Hypertension Patients Treated with GLP‐1 Receptor Agonists
Source: Ann Clin Transl Neurol. 2026 Apr 17:10.1002/acn3.70406. Online ahead of print. doi: 10.1002/acn3.70406 (PMC13395034; doi:10.1002/acn3.70406)
Supplement: Supplementary file 1 — Figure S1: Love plot for covariate balance before and after propensity score matching. [file ACN3-9999-0-s003.pdf]

Covariate Balance Before and After Propensity Score Matching

Standardized Mean Differences (SMD) — GLP-1 RA vs No GLP-1 RA in IIH Patients (N = 31,134 matched pairs)

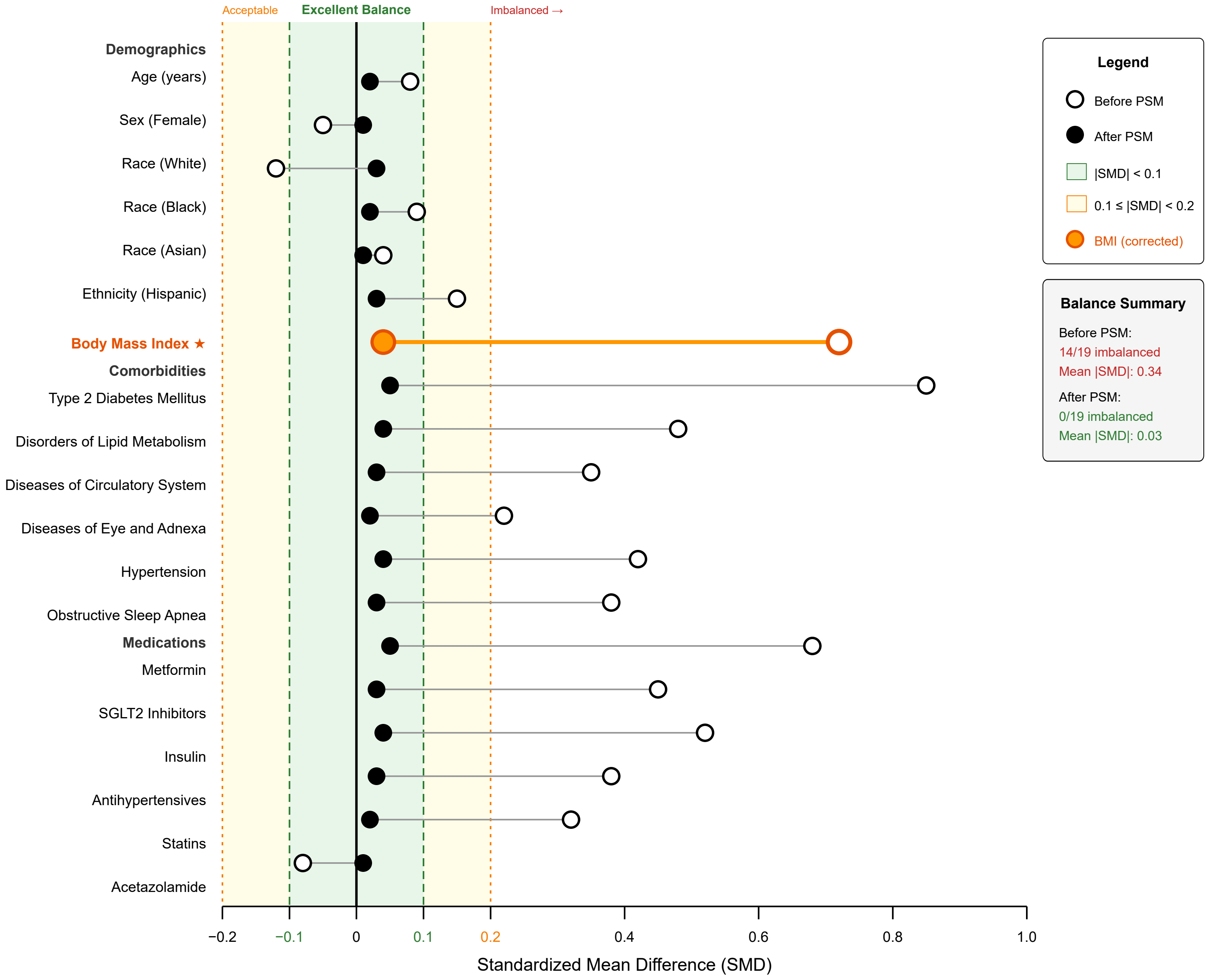

SMD = Standardized Mean Difference; PSM = Propensity Score Matching. Positive SMD indicates higher prevalence in GLP-1 RA group.

Balance thresholds: |SMD| < 0.1 indicates excellent balance (green zone); |SMD| < 0.2 indicates acceptable balance (yellow zone).

★ BMI correction achieved via exact matching on BMI category (normal/overweight/obese I/II/III) with 0.1 SD caliper on propensity score.
