# Supplementary material for: Risk of Non‐Arteritic Anterior Ischemic Optic Neuropathy in Idiopathic Intracranial Hypertension Patients Treated with GLP‐1 Receptor Agonists
Source: Ann Clin Transl Neurol. 2026 Apr 17:10.1002/acn3.70406. Online ahead of print. doi: 10.1002/acn3.70406 (PMC13395034; doi:10.1002/acn3.70406)
Supplement: Supplementary file 2 — Figure S2: Multiple testing correction plot. [file ACN3-9999-0-s001.pdf]

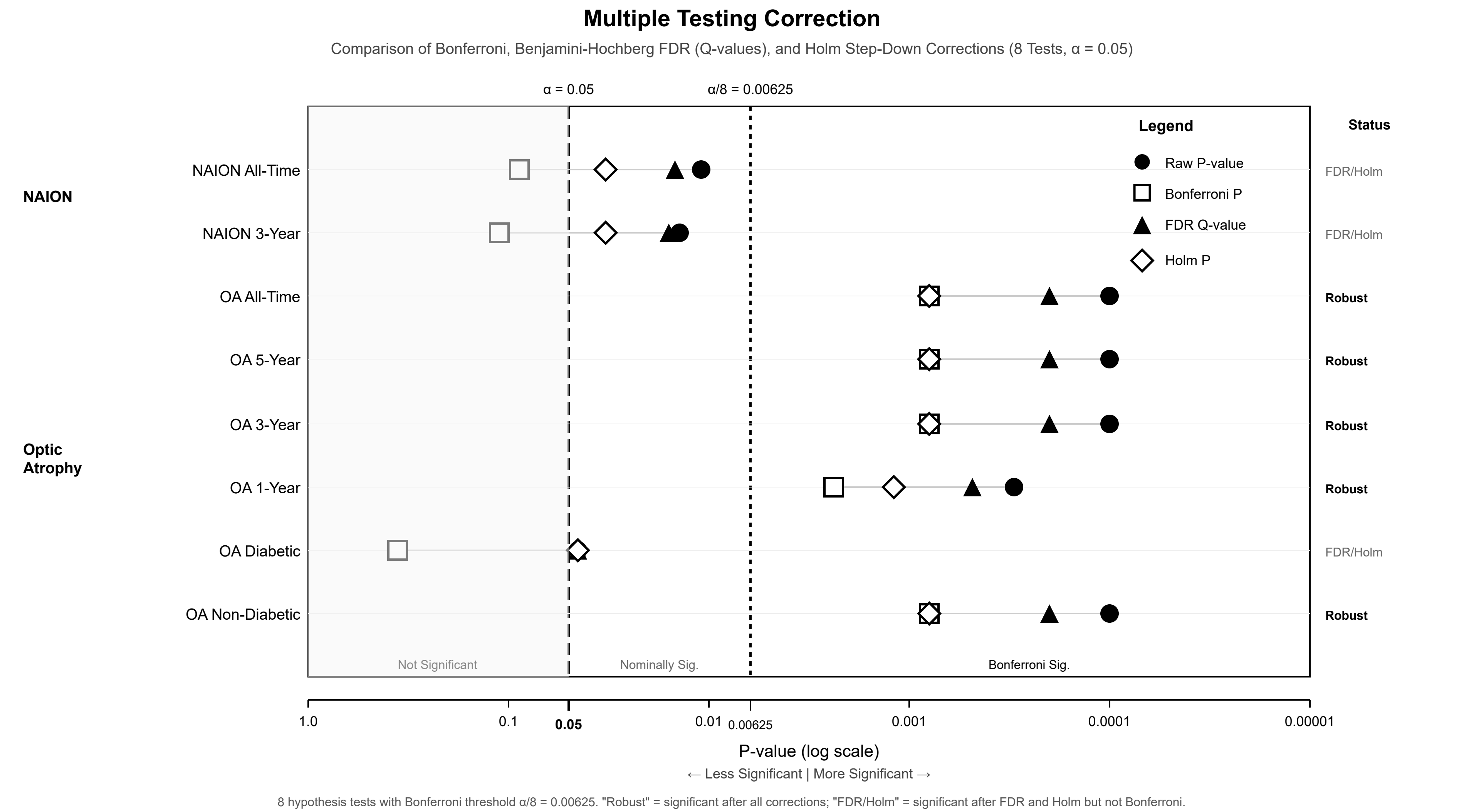

Multiple Testing Correction
Comparison of Bonferroni, Benjamini-Hochberg FDR (Q-values), and Holm Step-Down Corrections (8 Tests, α = 0.05)
α = 0.05
α/8 = 0.00625
Legend
Raw P-value
Bonferroni P
FDR Q-value
Holm P
Status
NAION
NAION All-Time
NAION 3-Year
Optic Atrophy
OA All-Time
OA 5-Year
OA 3-Year
OA 1-Year
OA Diabetic
OA Non-Diabetic
FDR/Holm
FDR/Holm
Robust
Robust
Robust
Robust
FDR/Holm
Robust
Not Significant
Nominally Sig.
Bonferroni Sig.
1.0
0.1
0.05
0.01
0.00625
0.001
0.0001
0.00001
P-value (log scale)
← Less Significant | More Significant →
8 hypothesis tests with Bonferroni threshold α/8 = 0.00625. "Robust" = significant after all corrections; "FDR/Holm" = significant after FDR and Holm but not Bonferroni.
